# Epstein–Barr virus-associated inflammatory pseudotumor variant of follicular dendritic cell sarcoma of the liver: a case report and review of the literature

**DOI:** 10.1186/s40792-022-01572-w

**Published:** 2022-12-09

**Authors:** K. Abe, M. Kitago, S. Matsuda, M. Shinoda, H. Yagi, Y. Abe, G. Oshima, S. Hori, Y. Endo, T. Yokose, E. Miura, N. Kubota, A. Ueno, Y. Masugi, H. Ojima, M. Sakamoto, Y. Kitagawa

**Affiliations:** 1 Department of Surgery, Keio University School of Medicine, Shinanomachi 35, Shinjuku-Ku, Tokyo, 160-8582 Japan; 2 Department of Pathology, Keio University School of Medicine, Tokyo, Japan

**Keywords:** Follicular dendritic cell sarcoma, Inflammatory pseudotumor variant, Epstein–Barr virus-associated tumor, Clinicopathological features, Molecular mechanisms, Laparoscopic surgery

## Abstract

**Background:**

Follicular dendritic cell sarcoma is a rare stromal tumor with no standard treatment. However, some reports have revealed that follicular dendritic cell sarcoma has an inflammatory pseudotumor variant associated with Epstein–Barr virus infection that has a relatively good prognosis. In this report, we present a case of a resected inflammatory pseudotumor variant of follicular dendritic cell sarcoma of the liver, and have reviewed the literature on the clinicopathological, molecular, and genomic features of this tumor.

**Case presentation:**

The inflammatory pseudotumor variant of follicular dendritic cell sarcoma originates only in the liver or spleen, causes no symptoms, and is more common in middle-aged Asian women. It has no characteristic imaging features, which partially explains why the inflammatory pseudotumor variant of follicular dendritic cell sarcoma is difficult to diagnose. Pathologically, the inflammatory pseudotumor variant of follicular dendritic cell sarcoma has spindle cells mixed with inflammatory cells and is variably positive for follicular dendritic cell markers (CD21, CD23, and CD35) and Epstein–Barr virus-encoded RNA. On genetic analysis, patients with this tumor high levels of *latent membrane protein 1* gene expression and extremely low levels of host *C–X–C Chemokine Receptor type 7* gene expression, indicating that the inflammatory pseudotumor variant of follicular dendritic cell sarcoma has a latent Epstein–Barr virus type 2 infection.

**Conclusions:**

The inflammatory pseudotumor variant of follicular dendritic cell sarcoma is an Epstein–Barr virus-associated tumor and a favorable prognosis by surgical resection, similar to Epstein–Barr virus-associated gastric cancer.

**Supplementary Information:**

The online version contains supplementary material available at 10.1186/s40792-022-01572-w.

## Background

Follicular dendritic cells (FDCs), also known as dendritic reticulum cells, are located primarily in the germinal centers of primary or secondary lymphoid follicles in nodal and extra-nodal sites. FDCs are derived from mesenchymal precursors; thus, they can become sarcomas but not carcinomas or lymphomas [1]. Monda et al. first described this tumor in 1986 based on a series of four cases that originated in lymph nodes [2]. Previous reports have pointed out that follicular dendritic cell sarcoma (FDCS) is an aggressive tumor with a high recurrence and metastasis rate [3]. FDCS mainly occurs in lymph nodes; however, approximately half of FDCS cases were found to occur in other sites, such as the liver, spleen, and small intestine. We have no standard treatment for this sarcoma, not only because of its rare incidence and the lack of therapies targeted toward it.

A recent report revealed a new subtype of FDCS that is the inflammatory pseudotumor (IPT) variant of FDCS that occurs only in the liver and the spleen and has a more favorable prognosis by resection than the “conventional” FDCS [4]. Moreover, the IPT variant of FDCS is associated with Epstein–Barr virus (EBV) infection, while conventional FDCS is not associated with EBV infection.

EBV is a gamma herpes virus composed of a linear double-stranded DNA (170–180 kilo-base-pair in size). EBV also contributes to the development of human cancers of epithelial, mesenchymal, and lymphocytic origins, because *EBV* gene products which are expressed in its latent infection, such as latent membrane protein (LMP) or EBV nuclear antigen (EBNA), are considered to be viral oncogenes [5]. These tumors also have various malignant natures with different prognoses; however, there is no antiviral drug or molecular-targeted therapy for EBV-associated tumors because of the lack of information regarding the relationship between EBV infection and oncogenesis.

In this report, we have presented the case of a patient in whom an IPT variant of FDCS of the liver was resected using laparoscopic surgery, and review the literature on the clinicopathological features of this variant and the underlying molecular mechanisms for oncogenesis.

## Case presentation

### Clinical presentation

**Fig. 1:**
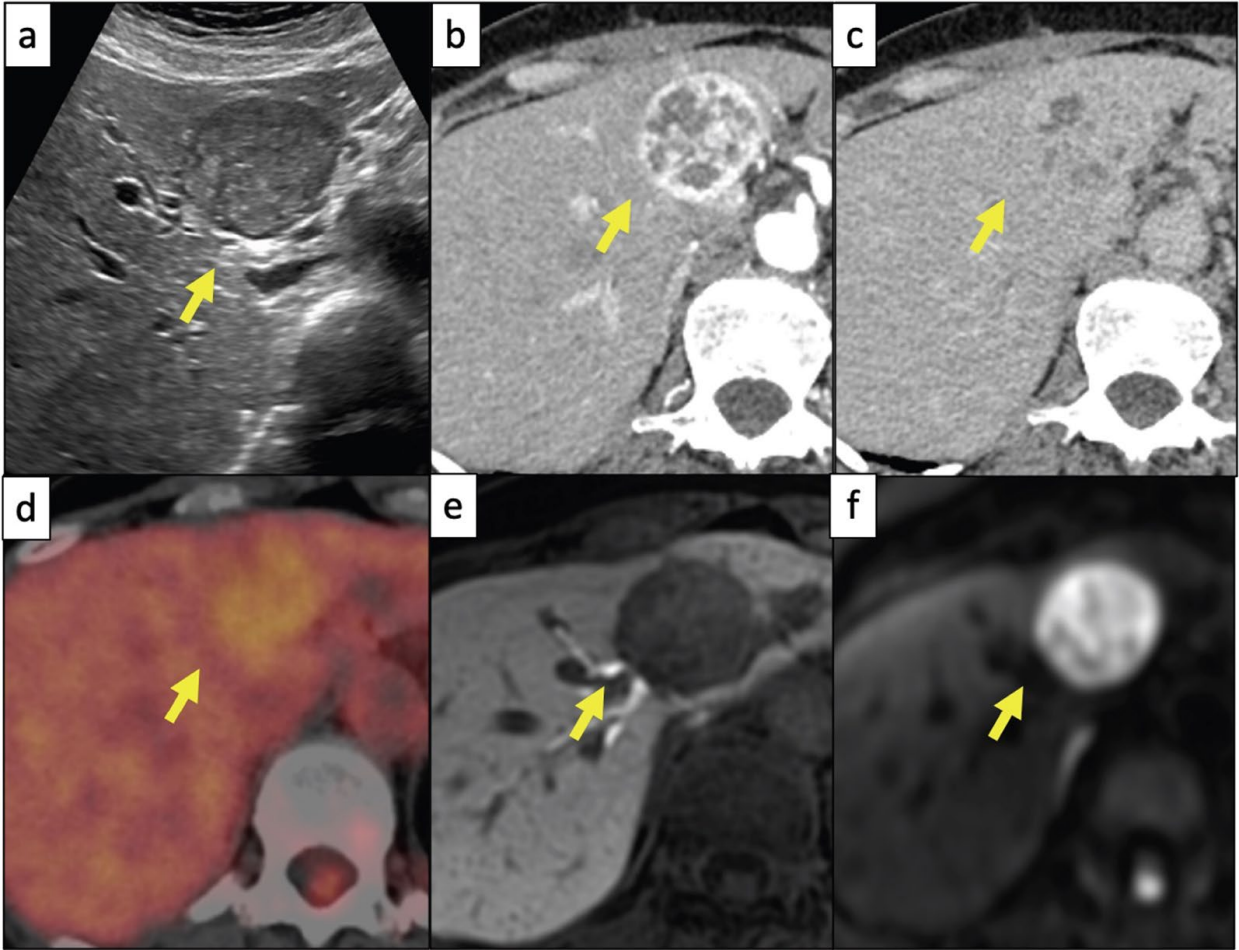
Preoperative imaging of a tumor. **a** Abdominal ultrasound showing a 4-cm mass in liver segment IV. **b** Tumor is heterogeneously enhanced with peripheral enhancement in the arterial phase of dynamic CT. **c** Early enhancing area is iso-intense to the liver parenchyma in the equivalent phase of dynamic CT. **d** FDG–PET shows slight hyperaccumulation in the tumor. **e** Signal of the tumor is markedly lower than that of the liver parenchyma in the hepatobiliary phase of gadolinium–ethoxybenzyl-diethylenetriamine penta acetic acid-enhanced MRI. **f** High signal intensity on diffusion-weighted image (*b* value of 1000 s/mm^2^) is noted. CT: computed tomography; FDG–PET: fluorodeoxyglucose–positron emission tomography; MRI: magnetic resonance imaging

**Fig. 2:**
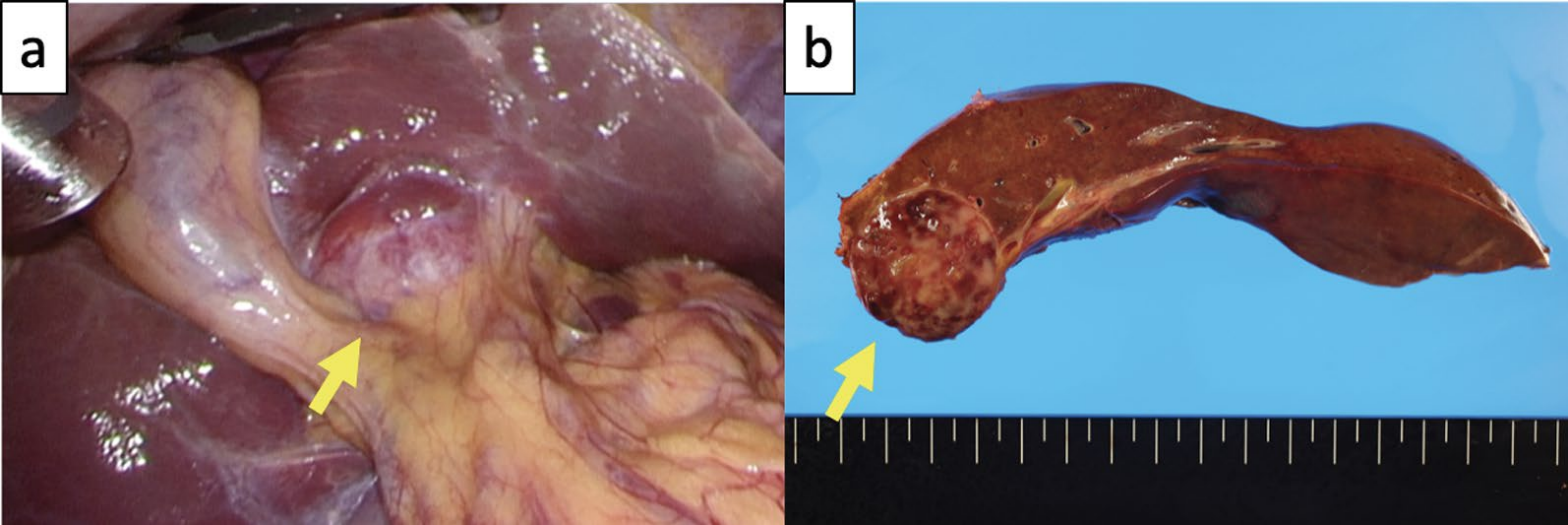
Intraoperative and macroscopic findings of EBV-associated FDCS. **a** Case underwent laparoscopic extended left hepatectomy (yellow arrow). **b** Mass measuring 3.5 cm in size, with a well-circumscribed margin, milky-white in color, and with necrotic components is noted. EBV: Epstein–Barr virus; FDCS: follicular dendritic cell sarcoma

A 60-year-old asymptomatic woman presented to our hospital with a 4-cm tumor in segment IV of her liver detected incidentally during a routine medical checkup. Laboratory investigations showed normal liver function test results, no viral infection, and no elevated tumor markers. Abdominal ultrasound revealed a 4-cm solitary, well-defined hypoechoic mass with mosaic components and flow signals in liver segment IV (Fig. 1a). The tumor was a heterogeneously enhancing lesion, with peripheral enhancement observed in the arterial phase of dynamic computed tomography (Fig. 1b). The early enhancing area appeared iso-intense to the liver parenchyma, as seen in the equivalent phase of dynamic computed tomography (Fig. 1c). The lesion also included several patchy areas without enhancement through all phases. Fluorodeoxyglucose–positron emission tomography showed slight hyperaccumulation in the tumor (Fig. 1d). In the hepatobiliary phase of gadolinium–ethoxy benzyl-diethylenetriamine penta-acetic acid-enhanced magnetic resonance imaging, the signal in the tumor was markedly lower than that in the liver parenchyma (Fig. 1e). A high signal intensity on a diffusion-weighted image (*b* value of 1000 s/mm^2^) was obtained (Fig. 1f). Laparoscopic extended left hepatectomy with intraoperative cholangiography was performed as the tumor had extended to the middle hepatic vein and left Gleason sheath (Fig. 2a). There were no complications during the postoperative course, and the patient was discharged from the hospital on postoperative day 8. She experienced no relapse for 3 years and 6 months.

### Pathological findings and final diagnosis

**Fig. 3:**
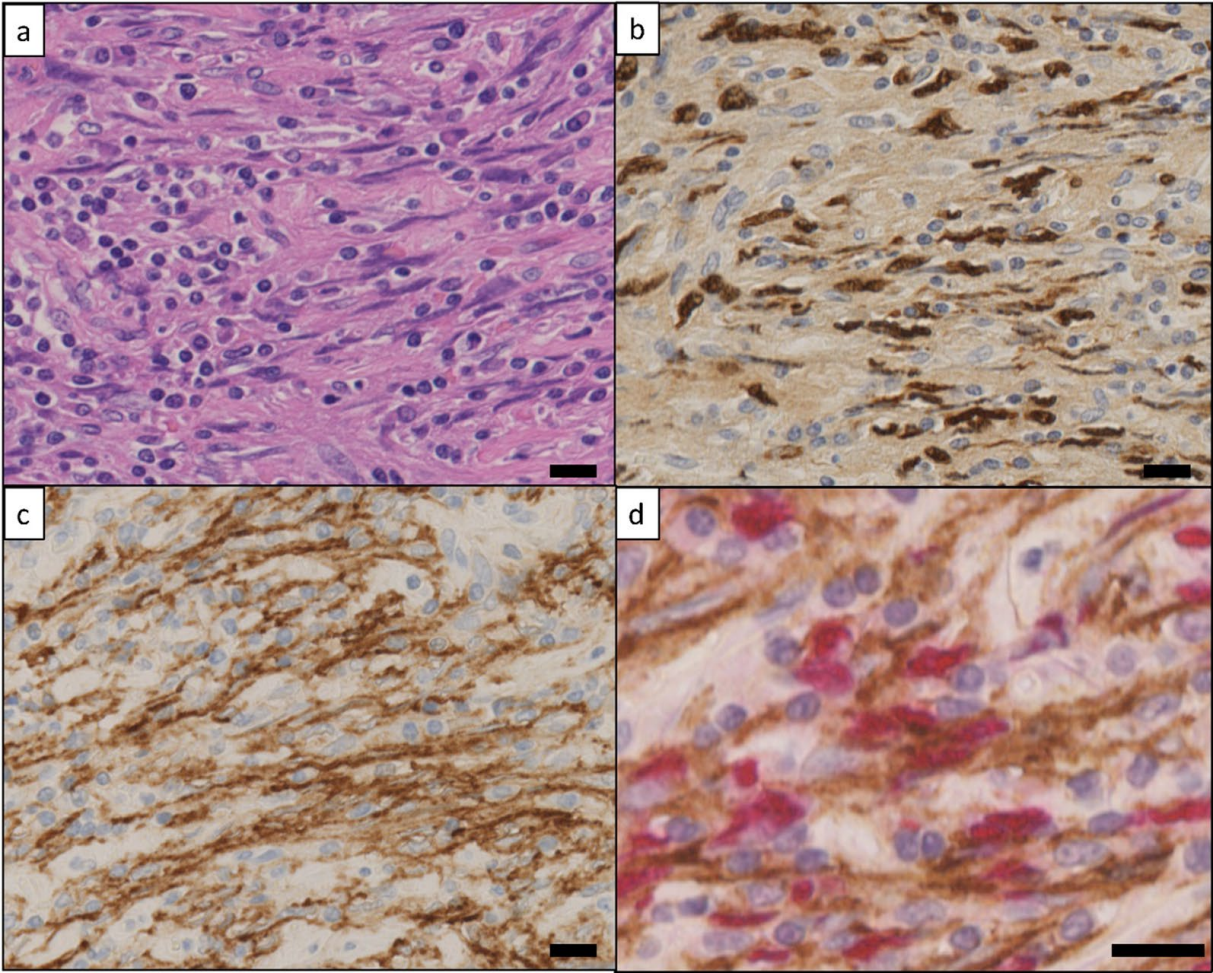
EBER–ISH and immunohistochemistry for EBV-associated FDCS (scale bars = 25 µm). **a** Lesion is composed of atypical spindle cells admixed with infiltrating inflammatory cells on hematoxylin and eosin staining. **b** Nucleus of the spindle cells is diffusely positive for EBER–ISH. **c** Cytoplasmic positivity for CD23. **d** Double staining for CD23 immunohistochemistry (brown) and EBER–ISH (red) revealing that EBER–ISH positive cells show CD23 immunoreactive cytoplasm. EBER: Epstein–Barr virus enucleated RNA; ISH: in-situ hybridization; FDCS: follicular dendritic cell sarcoma

A 3.5-cm-sized mass was recognized with a well-circumscribed margin, milky-white color, and necrotic components, as shown in Fig. 2b. Microscopically, the lesion was composed of atypical spindle cells mixed with inflammatory cells, chiefly lymphocytes, plasma cells, histiocytes, and a few neutrophils (Fig. 3a). These spindle cells showed positive nuclear signals for EBV-encoded RNA (EBER)–in-situ hybridization (ISH) (Fig. 3b). The tumor cells were positive for CD23 (Fig. 3c) and negative for CD79α, CD3, CD5, CD35, D2–40, and anaplastic lymphoma kinase (ALK). Immunoglobulin G4 (IgG4) positive cells were identified to constitute less than 1%. Double staining for CD23 and EBER further revealed that EBV-infected FDCs had the capacity for tumorigenesis (Fig. 3d). The Ki-67 level was approximately 5% in this tumor (Additional file 1: Fig. S1).

The mass had the following characteristics: IPT variant of FDCS (EBV-positive follicular dendritic cell sarcoma), size of 35 × 32 × 31 mm, simple nodular type, expansive growth, formation of capsule positive, Infiltration to capsule negative, septal formation negative, n0, vp0, vv2 (left hepatic vein adventitia), va0, b0, im0, surgical margin negative, T3 N0 M0, and Stage III (Union for International Cancer Control 8th edition).

### Genetic analysis

#### DNA sequence and single nucleotide polymorphisms of the IPT variant of FDCS

We obtained 900–1000-bp DNA fragments from the EBV-associated (IPT variant) FDCS using electrophoresis (Additional file 1: Fig. S2a). We extracted the DNA fragments and compared the sequence data with type 1 and 2 EBV genomes. The DNA fragments from the EBV-associated FDCS were 97% and 66% identical to the DNA of type 1 and type 2 EBV, respectively (Additional file 1: Fig. S2b).

Next, we performed multiple sequence alignments on the *RPMS1* gene of EBV (Additional file 1: Fig. S3). Annotation of the DNA sequence revealed that single nucleotide polymorphisms (SNPs) C155389T and G155391A were not present on the *RPMS1* gene in our case [6]. Moreover, in our case, the EBV DNA was identical to that isolated from B95-8 (lymphoblastoid cell line) and YCCEL1 (Korean gastric adenocarcinoma) but showed no similarity with the DNA isolated from GD2 and HKNPC1 (Chinese nasopharyngeal carcinomas [NPCs]) for SNP G155391A and from GD1 (Chinese NPC), Mutu (Kenyan Burkitt’s lymphoma), and AG876 (Ghanaian Burkitt’s lymphoma) for SNP C155389T.

#### LMP-1 and EBNA-2 (C–X–C Chemokine Receptor type 7 [CXCR7] in host cells) gene expression of the IPT variant of FDCS

**Fig. 4:**
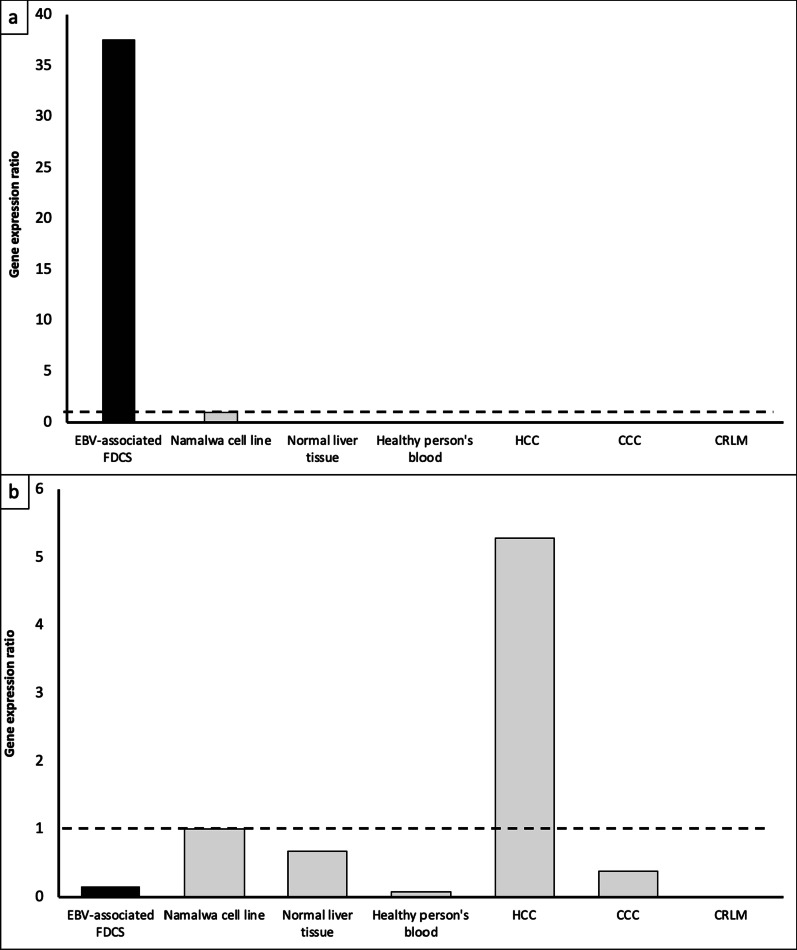
Real-time PCR analysis for gene expression. **a** PCR products were analyzed using the ΔΔCt method, and the *LMP-1* gene expression ratios in the tumor samples were calculated by setting the ratio for Namalwa cell line as 1. **b** PCR products were analyzed using the ΔΔCt method, and the *EBNA-2* (human *CXCR7*) gene expression ratios in the tumor samples were calculated by setting the ratio for Namalwa cell line as 1. EBV: Epstein–Barr virus; FDCS: follicular dendritic cell sarcoma; HCC: hepatocellular carcinoma; CCC: cholangiocellular carcinoma; CRLM: colorectal liver metastasis; PCR: polymerase chain reaction; LMP: latent membrane protein; EBNA-2: EBV nuclear antigen-2; CXCR7: C–X–C Chemokine Receptor type 7

EBV-associated tumors are subdivided into three latency types. Latency type I is expressed in gastric carcinoma, NPC, and Burkitt’s lymphoma. Latency type II corresponds to Hodgkin’s lymphoma and undifferentiated NPCs. Finally, cells from lymphoproliferative diseases arising in immunosuppressed hosts (e.g., patients with post-transplant lymphoproliferative disease and immunoblastic non-Hodgkin’s lymphoma) belong to latency type III [7, 8]. In previous reports, the *LMP-1* gene was positive in 90% of cases with the IPT variant of FDCS [9]; thus, we considered that the IPT variant of FDCS occurs in the latency type II or III. The level of *LMP-1* expression in this tumor was found to be 37.64 times that of the Namalwa cell line (Fig. 4a). In contrast, the level of *EBNA-2* (*CXCR7* in host cells) expression in this tumor was much lower than that of the Namalwa cell line and normal liver tissue (Fig. 4b). We also found that hepatocellular carcinoma (HCC) had a more than five times higher *EBNA-2* expression than the Namalwa cell line did. Besides, immunohistochemistry of LMP-1 and EBNA-2 revealed that this tumor had a high expression of LMP-1, but negative expression of EBNA-2 (Additional file 1: Fig. S4).

## Discussion

### Epidemiology and clinical features

FDCs are stromal-derived cells that are important for the maintenance of the architecture of B cell follicles within lymph nodes and other secondary lymphoid organs [1]. Hence, they are derived from mesenchymal stem cells, not from myeloid stem cells. Therefore, the tumorigenesis of FDCs indicates the formation of a sarcoma [6]. Conventional FDCS of the liver is a rare neoplasm that usually presents clinically with abdominal pain and weight loss [10]. In general, conventional FDCS presents in various locations, including within lymph nodes (31%) and outside them (58%) [11]. Furthermore, conventional FDCS has a high rate of recurrence (44.6%) and metastasis with a poor prognosis [11].

The IPT variant of FDCS of the liver is even rarer, and the clinicopathological features of this type remain unknown [12]. The IPT variant of FDCS of the liver has been reported in only 30 cases worldwide, including this case (Table 1; our case is the 30th case) [4, 13–32]. According to these reports, the IPT variant of FDCS of the liver emerged predominantly in women (*n* = 24, 80%) and in middle-aged patients whose median age was 48.5 (19–82) years. Furthermore, the IPT variant of FDCS of the liver had a favorable prognosis and low recurrence rate (five out of 30 cases, 16.7%). Recurrence sites were reported to be the liver in two patients and intraabdominal dissemination in one patient (the location in the two remaining patients was not described). Finally, the most significant difference between the IPT variant and conventional FDCS is the existence of EBV infection.

**Table 1:** Literature review of EBV-associated FDCS of the liver

No	Year published	Author	Gender	Age (years old)	Treatment	Follow-up (month)	Outcome
1	1996	Selves J [13]	Female	68	Chemotherapy + tumorectomy	30	No recurrence
2	1996	Shek TW [14]	Female	35	Right hepatectomy	95	Recurrence
3	1998	Shek TW [15]	Male	37	Right trisegmectomy + S1 resection	42	No recurrence
4	2001	Chen TC [16]	Female	57	Refusion	36	Alive
5	2001	Chen TC [16]	Female	51	Left hepatectomy	12	No recurrence
6	2001	Cheuk W [4]	Female	19	Tumorectomy	40	No recurrence
7	2001	Cheuk W [4]	Female	56	Right hepatectomy	56	Recurrence
8	2001	Cheuk W [4]	Female	40	Left hepatectomy	108	Recurrence
9	2001	Cheuk W [4]	Female	49	Tumorectomy	9	No recurrence
10	2001	Cheuk W [4]	Female	31	Right hepatectomy	60	No recurrence
11	2005	Torres U [17]	Male	82	Right hepatectomy	18	No recurrence
12	2006	Bai LY [18]	Female	30	Right hepatectomy	24	No recurrence
13	2007	Yuan J [19]	Male	29	Lateral segmentectomy	8	Recurrence
14	2007	Tu XY [20]	Female	28	Tumorectomy	ND	No recurrence
15	2008	Granados R [21]	Female	57	Tumorectomy	24	No recurrence
16	2008	Zhang ZX [22]	Male	40	Tumorectomy	3	No recurrence
17	2010	Liu Yh [23]	Female	59	Tumorectomy	17	No recurrence
18	2010	Zhang SH [24]	Male	75	Tumorectomy	6	No recurrence
19	2011	Xu B [25]	Female	50	Tumorectomy	6	No recurrence
20	2011	Martins PN [26]	Female	53	Left hepatectomy	6	No recurrence
21	2012	Tian BL [27]	Female	58	Right hepatectomy	30	No recurrence
22	2016	Chen Y [28]	Female	28	Left hepatectomy	48	Recurrence
23	2016	Chen Y [28]	Female	48	Extended right hemihepatectomy	23	No recurrence
24	2016	Chen Y [28]	Male	60	Wedge resection	3	No recurrence
25	2017	Zhang X [29]	Female	19	Segementectomy	12	No recurrence
26	2018	Endo Y [30]	Female	45	Left + S1 hepatectomy + PALN dissection	12	No recurrence
27	2019	Deng S [31]	Female	67	Radical right hepatectomy	ND	ND
28	2019	Zhang BI [32]	Female	48	Right hepatectomy	2	No recurrence
29	2019	Zhang BI [32]	Female	31	Laparoscopic right hepatectomy	10	No recurrence
30	2020	Our case	Female	60	Laparoscopic left radical hepatectomy	12	No recurrence

EBV: Epstein–Barr virus; FDCS: follicular dendritic cell sarcoma; PALN: paraaortic lymph node; ND: not described

### Imaging findings

The IPT variant of FDCS cannot be diagnosed by imaging modalities, mainly because of its various presentations and the lack of specific findings, as in this case where it could have been misdiagnosed as a neuroendocrine tumor of the liver, liver metastasis, or HCC. The IPT variant of FDCS shows a high fluorodeoxyglucose uptake, but fluorodeoxyglucose–positron emission tomography may not be useful for the differentiation of the IPT variant of FDCS from other tumors, such as metastatic tumors or lymphomas, as they can also show a high fluorodeoxyglucose uptake [33]. Based on the present and previous cases, we can conclude that the IPT variant of FDCS of the liver is a heterogeneous tumor with necrotic components that can expand and compress major hepatic vessels.

### Pathological findings

Since antigen loss is frequent, the diagnosis of FDCS requires support from immunohistochemistry, and the use of multiple FDC markers is often necessary. Tumor cells are variably positive for CD21, CD23, and CD35, which are specific markers of FDC [34, 35]. Liver IPT is a distinct disease that is often present on the differential with the IPT variant of FDCS. In the World Health Organization 2010 guidelines, liver IPT is subdivided into two types pathologically: IgG4-related and IgG4-negative, which are called the lymphoplasmacytic and fibrohistiocytic types, respectively [36]. The fibrohistiocytic type of IPT is frequently misdiagnosed as inflammatory myofibroblastic tumor, which is associated with ALK [37, 38]. Therefore, to diagnose the IPT variant of FDCS correctly, we must perform extensive immunohistochemical staining and recognize real tumor cells as positive for EBV markers (EBER–ISH, CD21, CD23, or CD35) and negative for IgG4 and ALK. From our results, we finally diagnosed that owing to the tumor cells, patients with the IPT variant of FDCS had an EBER-positive nucleus and a CD23-positive cytoplasm.

### Treatment strategies

Conventional FDCS can be cured by surgery alone but may recur many times with a poor prognosis after a long period. One review of conventional FDCS revealed that local recurrences and distant metastases were observed in 28% and 27% of cases, respectively [11]. The main sites of metastasis are the lung, liver, lymph nodes, and bones. This review also referred to negative predictive factors, such as tumor size (≥ 6 cm) and lymphoplasmacytic infiltration in tumor tissue [11]. Previous reports have insisted that there were no adjuvant treatments of significant effect on disease-free survival after surgical resection. Although, the IPT variant of FDCS has a more favorable prognosis than conventional FDCS, but the recurrence rate is estimated to be 16.7%; therefore, surgery with complete resection is the mainstay of treatment. In the present case, we performed laparoscopic hepatectomy that resulted in no complications or recurrence. Laparoscopic hepatectomy has been recently adopted for liver tumors, suggesting that laparoscopic resection of the IPT variant of FDCS might be feasible and safe for the short- and long-term outcomes of patients.

### EBV-associated tumors

Several types of EBV-associated tumors have been identified globally. First, EBV-associated gastric carcinoma (EBVaGC) constitutes approximately 10% of gastric carcinomas worldwide and is mainly observed in Asian countries [39], as EBV genomes discovered in EBVaGC are closely related to Asian-derived EBV strains, such as GD1 and 2, C666-1 (Chinese strain), or Akata (Japanese strain) [40, 41]. In addition, NPC is an EBV-associated epithelial carcinoma and is common in South China and Southeast Asia. More than 97% of NPC cases are caused by EBV infections, such as HKNPC 1 to 9. These Asian-derived EBV strains are categorized as type 1 EBV. In contrast, African Burkitt’s lymphoma is the most common in equatorial Africa and is designated as “endemic Burkitt’s lymphoma,” which is mainly caused by type 2 EBV [42]. Our research revealed that the IPT variant of FDCS in our case was derived from type 1 EBV, which was similar to the EBVaGC strain but different from NPC.

### Genetic findings

**Fig. 5:**
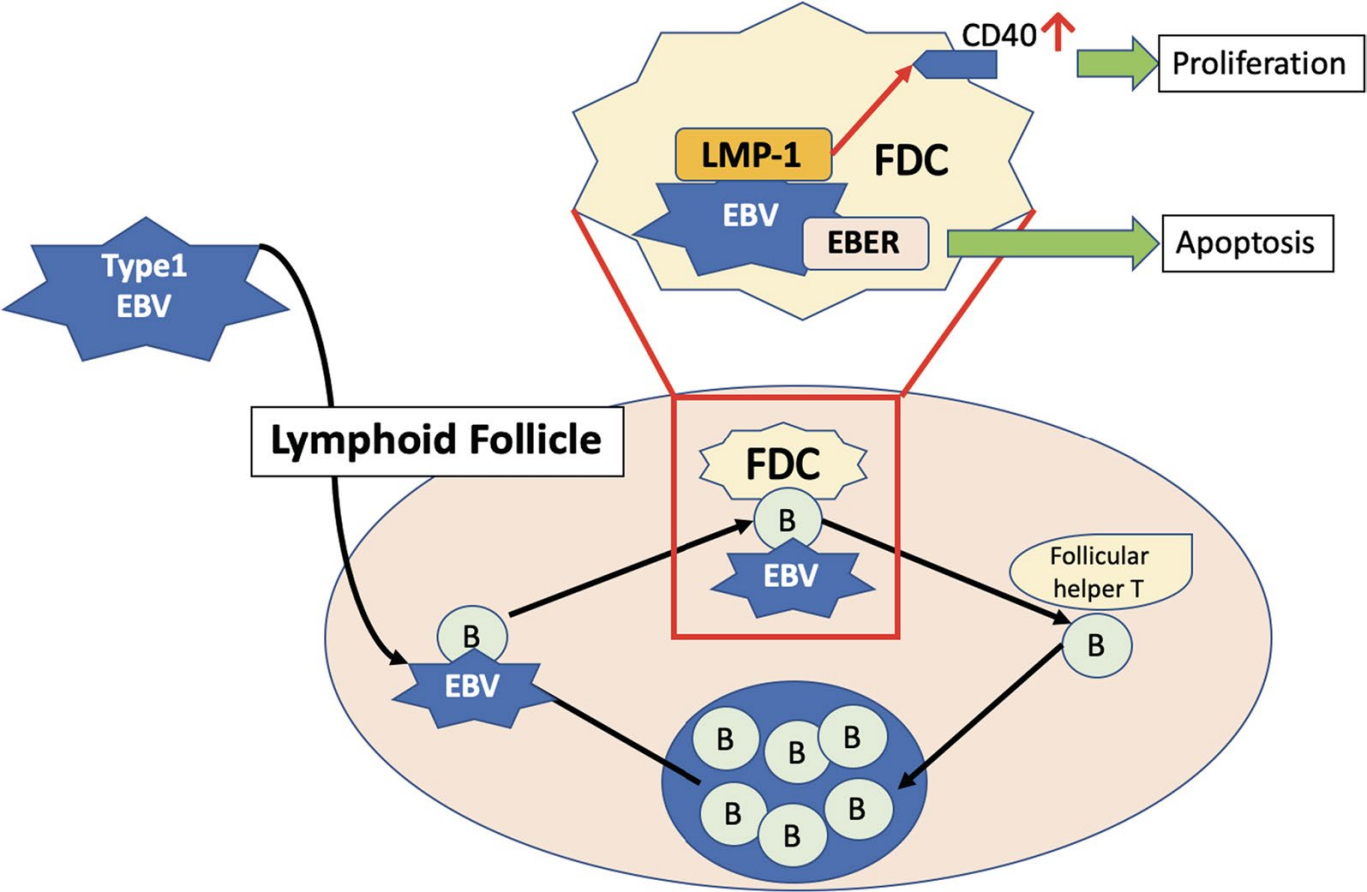
Oncogenesis of EBV-associated FDCS. Type 1 EBV initially infects human B lymphocytes and converts into the latency type. Then, it moves and infects the FDCs. EBER or LMP-1 protein released from the EBV to FDCs causes inhibition of cell apoptosis and proliferation of FDCs by amplification of the CD40 signaling pathways. IPT: inflammatory pseudotumor; FDCS: follicular dendritic cell sarcoma; EBV: Epstein–Barr virus; LMP-1: latent membrane protein-1; EBER: Epstein–Barr virus enucleated RNA

Based on our experience with this case, we propose the mechanism of tumorigenesis for FDCS associated with EBV infection (Fig. 5). Type 1 EBV initially infects human B lymphocytes in the lymphoid follicles, which generate in the liver tissue and are similar to the germinal center in the spleen [43, 44]. Then, EBV becomes latent in B cells and starts infecting FDCs. EBER or LMP-1 protein is released from EBV to FDCs, which causes the inhibition of cell apoptosis and proliferation of FDCs by amplifying CD40 signaling pathways. Chen et al. supported the finding that LMP-1 was elevated in cases of IPT-variant FDCS [16]. This mechanism of oncogenesis is similar to Hodgkin lymphoma associated with EBV infection. The latter is attributed to the crosstalk between EBV-infected B cells and follicular helper T cells in the light zone of the germinal center and the proliferation of B cells induced by EBER and LMP-1 proteins [45]. We also found that *EBNA-2* expression (*CXCR7* expression in host cells) was much lower in IPT-variant FDCS than in the Namalwa cell line, while that in HCC was much higher than that in the Namalwa cell line. *CXCR7* was reported to be overexpressed in various types of tumor endothelial cells, and the downregulation of *CXCR7* significantly inhibited the migration and invasion of HCC [46, 47], suggesting that the use of drugs that target this receptor is a promising treatment options in the future [48]. It is also curious about how blood levels of LMP-1 or CXCR-7 in this patient, which will lead to future preoperative diagnosis tool of this rare tumor.

### Future challenging issues

Despite the development of treatment strategy for various kinds of tumors, we still have several unresolved issues regarding IPT-variant FDCS. First, we do not know why EBV-positive solid tumors, including IPT-variant FDCS, have a relatively better prognosis than those without EBV association. Next, there is no targeted chemotherapy for FDCS including IPT-variant FDCS. Complete surgical resection remains the only treatment option. IPT-variant FDCS is one of the EBV-associated tumors; however, there is no targeted chemotherapy for EBV-associated tumors and no anti-EBV drugs, because EBV only infects humans and clinical trials for drug discovery against EBV infection have been delayed compared to those for other herpes viruses, such as simple herpes virus or varicella-zoster virus.

In summary, because of the very limited data, there are no definite treatment strategies, such as radiotherapy, chemotherapy, and immunotherapy, for FDCS and IPT-variant FDCS.

Although IPT-variant FDCS is a rare sarcoma associated with EBV infection, we hope that research on this rare tumor will lead to the discovery of the exact mechanism of tumorigenesis and help with future treatment options.

## Conclusions

IPT-variant FDCS is an EBV-associated tumor and may have a favorable prognosis following surgical resection, similar to EBV-associated gastric cancer.

## Supplementary Information

**Additional file 1.** Supplementary Figures and Tables.

## Data Availability

All data generated or analyzed during this study are included in this published article [and its supplementary information files].

## Abbreviations

FDCS: Follicular dendritic cell sarcoma
FDCs: Follicular dendritic cells
IPT: Inflammatory pseudotumor
EBV: Epstein–Barr virus
LMP: Latent membrane protein
CXCR7: *C–X–C Chemokine Receptor type 7*
EBNA: Epstein–Barr virus nuclear antigen
EBER: Epstein–Barr virus-encoded ribonucleic acid
ISH: In-situ hybridization
SNP: Single nucleotide polymorphisms
ALK: Anaplastic lymphoma kinase
NPC: Nasopharyngeal carcinomas
PCR: Polymerase chain reaction
HCC: Hepatocellular carcinoma
IgG4: Immunoglobulin G4
EBVaGC: Epstein–Barr virus-associated gastric carcinoma
