# Supplementary material for: Epstein–Barr virus-associated inflammatory pseudotumor variant of follicular dendritic cell sarcoma of the liver: a case report and review of the literature
Source: Surg Case Rep. 2022 Dec 9;8:220. doi: 10.1186/s40792-022-01572-w (PMC9733763; doi:10.1186/s40792-022-01572-w)
Supplement: Supplementary file 1 — Additional file 1. Supplementary Figures and Tables. [file 40792_2022_1572_MOESM1_ESM.docx]

**Figure S1. Ki-67 staining of IPT-variant FDCS (scale bars = 25 µm).** Ki-67 was about 5% of this tumor.

**
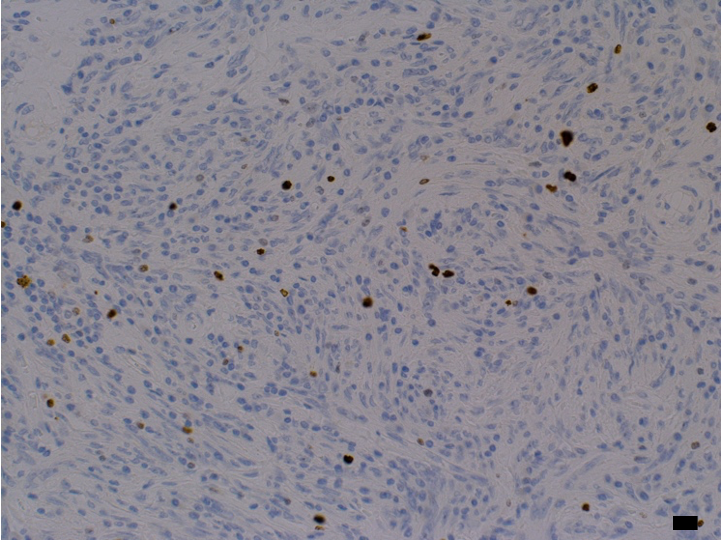
**

**Figure S2.** **DNA structure of the EBV obtained from IPT-variant FDCS.** a. Electrophoresis of PCR products was performed for normal liver tissue and IPT-variant FDCS. b. Sequence data of IPT-variant FDCS were compared with those of type 1 and type 2 EBV strains. IPT, inflammatory pseudotumor; FDCS, follicular dendritic cell sarcoma; EBV, Epstein–Barr virus; PCR, polymerase chain reaction.


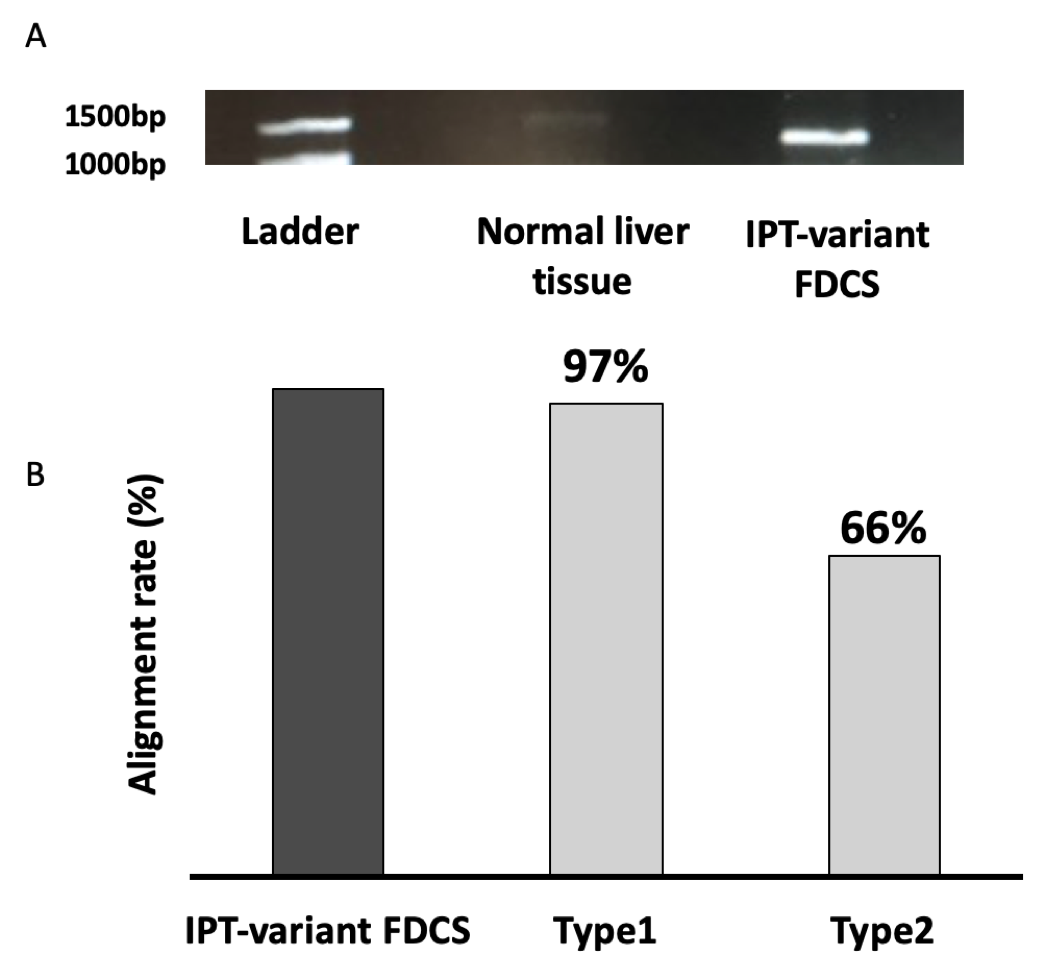


**Figure S3.** **Multiple sequence alignment in *RPMS1* gene region.** SNPs on *RPMS1* gene of our case turned out to be same as that of B95-8 (lymphoblastoid cell line) and YCCEL1 (Korean gastric adenocarcinoma), but different from that of Chinese NPCs (GD1,2, HKNPC1) and African Burkitt lymphomas (Mutu and AG876). SNP, single nucleotide polymorphism; NPC, nasopharyngeal carcinoma.

**Supplementary Figure 4. LMP-1 and EBNA-2 immunohistochemistry of IPT-variant FDCS (scale bars = 25 µm).**

1. LMP-1 was highly expressed in tumor tissue.


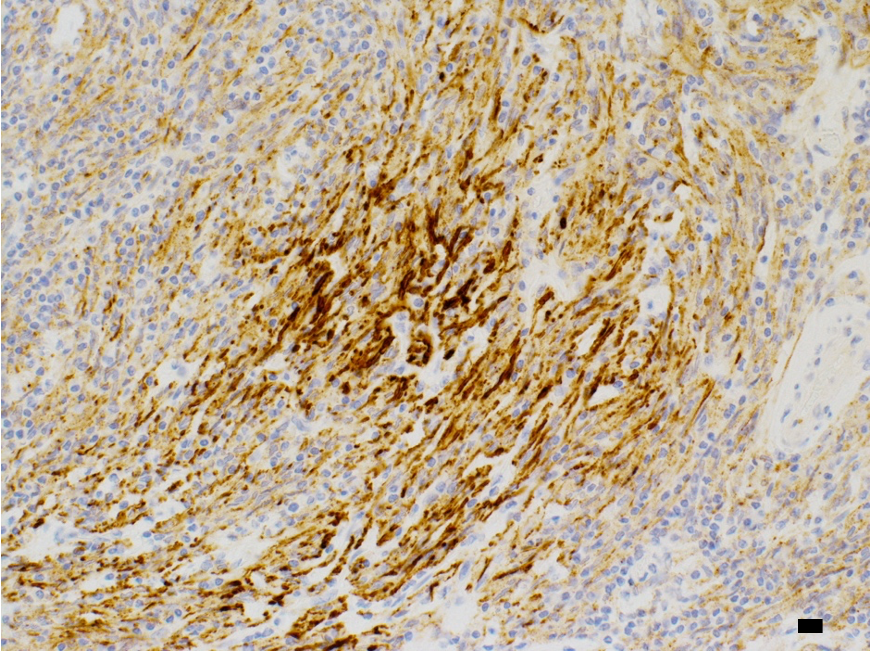


1. EBNA-2 was negative in tumor tissue.

**
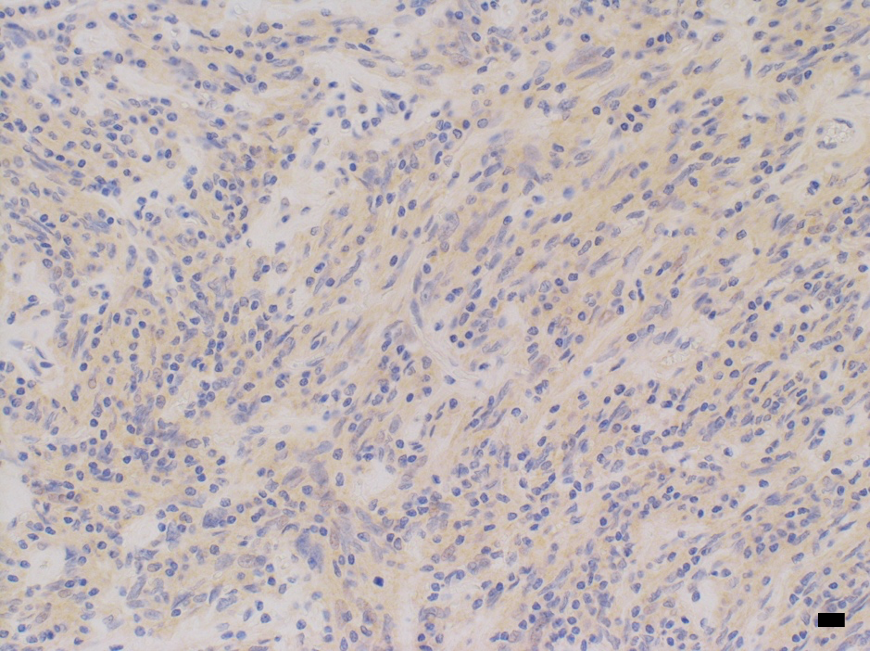
**

**Table S1.** List of antibodies used for immunohistochemistry

| No. | Antibody | Clone | Manufacturer | Positive control | Negative control | Validation | References |
| --- | --- | --- | --- | --- | --- | --- | --- |
| 1 | Anti-CD21 | 1F8;  dilution 1:200 | DAKO | B lymphocyte | Jurkat cell  (T-cell line) | Immuno-histochemistry | (8) |
| 2 | Anti-CD23 | 1B12;  dilution 1:200 | Leica  Microsysems | B-cell chronic lymphocytic leukemia | A431 cell (epidermoid carcinoma) | Immuno-histochemistry | (9) |
| 3 | Anti-CD35 | SP191;  dilution 1:100 | Abcam | Human colon and  tonsil tissues | Human dermal  fibroblast cells | Immuno-histochemistry | (10) |

**Table S2a.** Sequences of primers and TaqMan probes used for PCR

| *EBNA-2* | Forward | 5′- CAGGGATGCCTGGACACAAG -3′ |
| --- | --- | --- |
|  | Reverse | 5′- GGTTCATGTATTGGTGAAA -3′ |
|  | Amplicon size | 978 bp for type 1 EBV and 957 bp for type 2 EBV |
|  | Nucleotide # | 11305 to 37739 |
|  |  |  |
| *RPMS1* | Forward | 5′- TGTAGATGTGCCTGGCTCTG -3′ |
|  | Reverse | 5′- CCATCCCAACAGTGTGTGTC -3′ |
|  | Amplicon size | 565 bp |
|  | Nucleotide # | 138352 to 160531 |

**Table S2b.** Sequences of primers and TaqMan probes used for real-time PCR

| *LMP-1* | Forward | 5′- CAGTCAGGCAAGCCTATGA -3′ |
| --- | --- | --- |
|  | Reverse | 5′- CTGGTTCCGGTGGAGATGA -3′ |
|  | Probe | 5′- (6FAM) GTCATAGTAGCTTAGCTGAAC (TAMRA) -3′ |
|  | Amplicon size | 104 bp |
|  | Nucleotide # | 168117 to 168221 |
|  |  |  |
| *GAPDH* | Lot # | 446724 |
|  | Mastermix | Hs99999905_n1 |
|  |  |  |
| *ACKR3* | Lot # | 1736468 |
| *(CXCR7)* | Mastermix | Hs00664172_s1 |

**Materials and Methods**

**1. Immunohistochemistry and in-situ hybridization**

Immunohistochemistry and ISH were performed on 4-mm-thick, formalin-fixed, paraffin-embedded tissue sections. Each section was deparaffinized in xylene, rehydrated, and incubated in 0.03% H_2_O_2_ in 95% methanol for 10 minutes. The used antibodies and the heat-induced epitope retrieval methods were prepared at 120℃ for 5 minutes using an autoclave. We also used a Bond-Max automated staining machine (Leica Microsystems, Milton Keynes, UK). EBER-ISH studies were performed using a BOND EBER probe (Leica Microsystems). Immunohistochemistry was performed with anti-CD21 antibodies (clone 1F8; dilution 1:200; DAKO) and a heat-mediated antigen retrieval method (BOND enzyme pretreatment Kit pH6 for 10 minutes), anti-CD23 antibodies (clone 1B12, 1:200, Leica Microsystems) and a heat-mediated antigen retrieval method (citrate buffer pH6 for 30 minutes), and anti-CD35 antibodies (clone SP191, 1:100, Abcam) and a heat-mediated antigen retrieval method (EDTA buffer, pH9 for 40 minutes). We also performed double staining for CD23 and EBER. Immunohistochemistry for CD23 was first performed using 3,3’-diaminobenzidine (BOND Polymer Refine Detection, Leica Biosystems, Wetzlar, Germany) with brown staining, and then EBER-ISH was performed using Fast Red (BOND Polymer Refine Red Detection, Leica Biosystems) with red staining.

**2. Genetic analysis**

2.1. Analysis methods

We used resected frozen specimens retrieved from the IPT variant of FDCS. We used DNA and RNA from the Namalwa cell line (Japanese Cancer Research Resources Bank. Cell number IFO50040, #07182008) as a standard by which the EBV genome was measured. Glyceraldehyde-3-phosphate dehydrogenase was used as a house-keeping gene. DNA and RNA were extracted from the resected frozen specimens in the tumors, normal liver tissues, and normal blood using the DNeasy and RNeasy Blood and Tissue kits (QIAGEN®︎, Hilden, Germany) according to the manufacturer’s protocol. DNA and RNA concentrations were determined using a Nano-Drop spectrophotometer (Thermo Fisher Scientific, Wilmington, DE, USA), and we subsequently performed the reverse transcription from RNA to complementary DNA. The polymerase chain reaction (PCR) conditions were as follows: 94℃ for 2 minutes; 94℃ for 30 seconds, 55℃ for 30 seconds, and 72℃ for 90 seconds for 40 cycles; and 72℃ for 6 minutes, using TaqMan probes (Thermo Fisher Scientific®︎). Real-time PCR was performed, and products were detected using an Applied Biosystems ViiA™ 7 Real-Time PCR System (Thermo Fisher Scientific®︎). The thermocycling conditions were as follows: 50℃ for 2 minutes; 95℃ for 10 minutes; 95℃ for 15 seconds, and 60℃ for 1 minute for 40 cycles. We analyzed PCR products using ΔΔCt methods and calculated the expression ratios of tumors by setting the ratio for Namalwa cell line as 1.

2.2. EBV-DNA’s structure and single nucleotide polymorphisms of IPT-variant FDCS

We analyzed the EBV genome present within tumor cells. Type 1 and 2 EBV encode different *EBNA2* genes, with only 54% amino acid sequence similarity [5]; therefore, we extracted DNA and performed PCR for *EBNA2* gene. Next, we investigated SNPs of EBV genome because we could not perform PCA analysis with one specimen. We analyzed G155391A in the *RPMS1* open reading frame that has been correlated with a high incidence of NPC. PCR primers of *EBNA2* and *RPMS1* are shown in Table 1b. To gain high purity of PCR products, we performed electrophoresis using 1% agarose gel and extracted DNA cutting out the gel in the area of product size following QIAEX-II (QIAGEN®︎) protocols.

2.3. *LMP-1* and *EBNA-2* gene expression of the IPT variant of FDCS

To detect latency types, we performed gene expression analyses for *LMP-1* and *EBNA-2.* We assessed *EBNA-2* expression using *CXCR7* expression in tumor cells because viral EBNA-2 protein highly induced CXCR7 expression in host cells [12]. CXCR7 belongs to the member G protein-coupled receptor families. Therefore, RNA extraction and real-time PCR for *LMP-1* and *CXCR7* were performed. Real-time PCR probes are shown in Supplementary Table 1. We also used normal liver tissue retrieved from this patient, and we used Namalwa cell line as a positive control and one living blood donor as a negative control. Furthermore, we analyzed other types of liver tumors, such as HCC, cholangiocellular carcinoma, and metastatic liver tumor originating from colorectal cancer.
